# Social and Workplace Experiences of Individuals with a History of Cancer in Newfoundland and Labrador

**DOI:** 10.3390/curroncol33060356

**Published:** 2026-06-13

**Authors:** Krista King, Derrick Bishop, Stephanie Budgell, Melanie Vokey, Georgia Skardasi, Cindy Whitten, Teri Stuckless, Holly Etchegary, Sevtap Savas

**Affiliations:** 1 Division of Population Health and Applied Health Sciences, Faculty of Medicine, Memorial University of Newfoundland, St. John’s, NL A1B 3V6, Canada; krista.king@mun.ca (K.K.); holly.etchegary@mun.ca (H.E.); 2 Division of Biomedical Sciences, Faculty of Medicine, Memorial University of Newfoundland, St. John’s, NL A1B 3V6, Canada; 3 Department of Research and Innovation, Eastern Health, St. John’s, NL A1B 3V6, Canada; 4 Faculty of Humanities and Social Sciences, Department of Sociology, Memorial University of Newfoundland, St. John’s, NL A1C 5S7, Canada; 5 Discipline of Oncology, Faculty of Medicine, Memorial University of Newfoundland, St. John’s, NL A1B 3V6, Canada

**Keywords:** cancer, lived experience, Newfoundland and Labrador, patient-oriented research, survivorship, qualitative analysis

## Abstract

**Introduction:** As global cancer incidence and survival rates continue to rise, understanding the experiences and needs of individuals in the survivorship phase is critical to inform policies that promote equitable care and adequate support for cancer survivors. **Objective:** The objective of this study was to examine the lived social and workplace experiences of cancer survivors in Newfoundland and Labrador (Canada) using a qualitative research design. **Methods:** The study was open to cancer survivors of majority age who resided in Newfoundland and Labrador after their diagnosis. Between June 2023 and August 2024, twenty-five individuals participated in the study. Data were collected virtually through focus groups, individual interviews, or written responses. Demographic and socioeconomic characteristics of participants were collected via a survey. Thematic analysis was performed on all qualitative data. Two patient partner investigators informed the research throughout the entire project. **Results:** Data were rich and diverse, revealing a range of positive and negative experiences in social and workplace settings. Major themes included stigma in social and workplace environments, financial toxicity, workplace accommodations, social support and information needs. Young participants had unique challenges. Participants offered recommendations aimed at enhancing available supports and improving the quality of life of cancer survivors. Overall, findings highlight shared experiences across different regions and cultures while also painting the local context. **Discussion:** The results of this study reveal diverse experiences among cancer survivors within social and workplace settings. The findings and resulting recommendations can inform meaningful improvement to policies and programs, thus promoting equity and enhancing the lived experiences of cancer survivors.

## Simple Summary

In this study, we aimed to learn cancer survivors’ experiences in their social and workplace environments in a Canadian province. For this purpose, we had group discussions, interviews, or written correspondence with 25 people who have had cancer. Analysis of participant narratives revealed significant challenges associated with social support, access to information, financial support, and employment-related experiences. Participants provided recommendations to strengthen existing supports and improve survivorship experiences for future cancer survivors. Our findings align with existing literature and highlight the need for targeted policy initiatives, enhanced government and workplace support programs, and increased public awareness to better address the complex survivorship needs of cancer survivors.

## 1. Introduction

Due to significant advancements in the detection, diagnosis, and treatment of cancer, more individuals are living with or beyond a diagnosis of cancer than ever before. The International Agency of Research on Cancer [1] and World Health Organization [2] estimate that by 2050, there will be over 35 million new cancer cases worldwide, representing a 77% increase in new cancer cases since 2020 [2]. With a growing cancer burden worldwide, it is imperative to gain a comprehensive understanding of the impact of cancer and the supports required to meet the complex and ongoing needs of individuals, families, and communities affected by cancer.

Cancer survivors may experience numerous adverse effects stemming from both the disease and its treatment. These challenges can include impaired cognitive functioning, changes in sexual and reproductive health, disruptions in social relationships, financial strain, and impacts on emotional and psychosocial wellbeing, along with interruptions to educational and career development [3,4,5,6,7,8,9,10,11]. As a result, survivors may face reduced or lost workplace responsibilities, decreased income or employment opportunities, and challenges in securing appropriate academic or workplace accommodations [6,9,10].

The Institute of Medicine emphasized the importance of preventing and managing the impacts of cancer and its treatments, so that survivors’ ongoing needs can be met [12]. Unfortunately, cancer survivorship research is underfunded; as a result, cancer survivorship remains understudied compared to other areas of the cancer care continuum. From 2005 to 2021, cancer survivorship research represented only approximately 4% of overall cancer research funding in Canada [13].

Situated at the eastern edge of Canada, the province of Newfoundland and Labrador (NL) spans approximately 405,720 square kilometres and has a population of about 550,000 [14,15]. Despite higher per capita healthcare spending, the residents of NL experience poorer health outcomes than those in other provinces and have one of the highest burdens of cancer in Canada [16,17]. Geographic dispersion of the NL population creates significant structural and logistical challenges that may limit consistent access to timely screening, specialized oncology services, and supportive care services.

Strengthening cancer survivorship evidence is necessary to ensure that cancer survivors receive holistic, person-centered care that supports optimal health outcomes and enhanced quality of life. This study aimed to gain an in-depth understanding of the lived social and workplace experiences of cancer survivors in a Canadian province with a high rate of cancer. Our results show rich participant experiences both in the social and workplace dimensions that extend the existing literature on the topic, and illustrate the experiences of a population spread over a relatively large land. Findings can inform the development of better cancer care policies and the implementation of better support programs in this province and the rest of Canada.

## 2. Methods

### 2.1. Study Design

This study used a qualitative, patient-oriented approach to examine the lived social and workplace experiences of cancer survivors in NL. Two patient partners (SB, DB) contributed to the study’s design and provided input across its development, research, and dissemination stages.

### 2.2. Study Population

Participants were eligible for inclusion in this research study if they: (1) resided in NL; (2) were at least 18 years of age; (3) were diagnosed with cancer in the last five years (at the time of recruitment); (4) had full-time or part-time work experience following cancer diagnosis; (5) had access to a phone, internet, or computer; and (6) were able to provide informed consent. Twenty-five individuals (*n* = 25) who met the eligibility criteria and consented to participation were included in the study. Table 1 summarizes the sociodemographic characteristics of participants. Throughout this manuscript, we use the term “cancer survivor” to describe people who have a history of cancer diagnosis and are going through or have completed their cancer treatment.

### 2.3. Participant Recruitment

The study was promoted through multiple recruitment channels. Social media platforms were used, including paid advertisements on Facebook and posts on Twitter and LinkedIn. Announcements were made through Memorial University communications (Gazette; Newsline) and the provincial patient-oriented research support unit [19], as well as local media outlets. Study posters were displayed in community settings and circulated through community support organizations, including Young Adult Cancer Canada [20] and NL Candlelighters [21]. Collaboration with oncologists and cancer nurse navigators at cancer clinics across NL further supported study promotion. Additionally, 1000 study information letters were mailed to eligible survivors identified through the NL Cancer Care Registry. Study details were shared with registered nurses and nurse practitioners via the College of Registered Nurses of Newfoundland and Labrador’s practice newsletter. Finally, information about the study was made available on the study lead’s website [22]. Individuals who expressed interest in the study were directed to contact a research team member (KK) to obtain detailed information about the study. Eligible and consented participants could choose to participate in the study through a virtual focus group (FG), a one-on-one virtual individual interview (II) conducted via WebEx [23], or by submitting a written response (WR) via email, according to their availability and preferences. While FGs, WRs, and IIs may differ in their levels of ability to collect data, which may affect thematic analyses, they also provide flexibility and increase inclusivity and participation. The method of sampling was convenience sampling. It is possible that individuals who are more enthusiastic or comfortable discussing survivorship issues may have been more likely to participate in this study, which can create selection bias.

### 2.4. Data Collection

Data collection took place between 29 June 2023 and 8 August 2024. The primary method of data collection utilized in this study was virtual FGs. The design of the virtual FG sessions was guided by Krueger and Casey [24]. Initially, we aimed to have FGs from each of the four main health zones in NL (Eastern, Western, Central, and Labrador–Grenfell) to explore potential differences in participant experiences based on their location. Additionally, we aimed to have a specific FG for young adults residing in NL (18–45 years of age). This group was targeted as a previous study in the NL population showed that young survivors were at increased risk of stigma [25]. During the recruitment phase, the recruitment outside of the Eastern Health zone was limited. Hence, we opted to hold one FG including participants from Western, Central, and Labrador–Grenfell zones of the NL Health Services and two focus group sessions for the Eastern Health zone (this zone is the largest, serving approximately two-thirds of the population [15]).

FG sessions were conducted via the WebEx platform [23], vetted by Memorial University, and ranged in duration from 44 to 87 min. IIs lasted between 33 and 55 min. KK moderated the virtual discussions/interviews while SS observed. A semi-structured interview guide containing open-ended questions was used to guide discussions/interviews. Participants were reminded of the importance of protecting the privacy and confidentiality of information shared during the group sessions. At the end of the virtual sessions, participants were reminded of local resources that they could access for emotional support, if needed. Each virtual FG and II session was audio- and video recorded in WebEx. FG 1 included seven individuals from Eastern Health Zone; FG 2 consisted of four young adults; FG 3 included five individuals from across the province; and FG 4 additional four individuals from the Eastern Health Zone. In addition, two participants from the Eastern Zone elected to complete an II, and three participants opted to submit a WR, two of whom were young adults and one of whom was from the Eastern Zone. The focus group guide can be found in Supplementary Document S1. Prior to participation, participants completed a survey to collect information about their disease history as well as demographic and economic features (see Supplementary Document S2). Participants were offered a gift card in the amount of $25.

### 2.5. Data Analysis

The qualitative data obtained from virtual FGs and IIs were audio- and video-recorded. Team members KK, MV, and GS independently verified that their data were transcribed verbatim. KK analyzed the transcripts from the virtual FGs, IIs, and WRs using thematic analysis to identify key themes. A step-by-step approach to thematic analysis was implemented to enhance the rigour of the qualitative data analysis [26]: data familiarization, generating initial codes, searching for themes, reviewing themes, defining and naming themes, and producing the report. Initial themes were then shared with research team members in two meetings for further discussion. Following this, KK refined the themes. Sociodemographic survey data is presented as numbers and percentages. During this study, healthcare-related themes were also identified, which we plan to report in another article. Figures were created using Canva [27].

## 3. Results

A total of 25 individuals participated in the study (Table 1), representing diverse cancer diagnoses, with breast cancer (28%) being the most common. Almost two-thirds of participants resided in the Eastern Zone of NL Health Services, a region of the province with generally better access to health resources than other areas of the province. Most participants self-identified as women (72% women vs. 28% men) and White (80% White, 4% Indigenous, 4% Black–African, 4% Mixed ethnicity, and 8% undeclared).

### 3.1. Social Experiences

**Figure 1:**
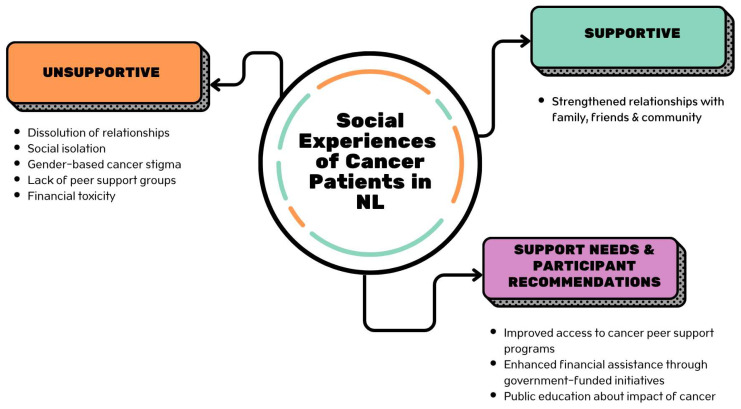
Social experiences.

Participants described a range of supportive and unsupportive lived social experiences following their cancer diagnosis (Figure 1). These reflections highlight the importance of meaningful social connections throughout the cancer journey.

#### 3.1.1. Supportive Social Experiences

Supportive social experiences included strengthened connections with family, friends, and the broader community. Participants described receiving social support through in-person visits, meal preparation and grocery delivery, messages and social media outreach, and community-organized fundraising initiatives. One participant reflected, *“My relationships with family and friends have changed for the better… It was very clear to me how much everyone cared about and supported me”* (WR 1).

Another participant stated, *“Kind notes, messages on Facebook, those things helped lift my spirits. I had breast cancer and I lost both my breasts, and that was really hard. I cried and cried and cried a lot. The visits, the kind gifts, and those sorts of gestures were what really helped me get through”* (FG 4).

#### 3.1.2. Unsupportive Social Experiences

**Dissolution of relationships.** Several participants described the deterioration or dissolution of significant relationships following their cancer diagnosis with partners, family members, and friends. One participant shared, “*As soon as I was diagnosed with breast cancer, my partner … advised me that he didn’t want to… have to go through any of that”* (FG 3). In some cases, these relational disruptions exacerbated the psychological burden of coping with cancer.

**Social isolation.** Some participants chose not to disclose their cancer diagnosis to others. This decision was often motivated by the need to maintain normalcy, to avoid stigma, or to protect others from worry. One participant shared, *“It was really difficult… you know, you’re carrying the secret, and I didn’t want anyone to know at all”* (FG 1). Non-disclosure often contributed to the feelings of loneliness and psychological distress.

**Gender-based cancer stigma.** Several participants diagnosed with cancers affecting female reproductive organs described experiences of gender-based cancer-related stigma. For some, the diagnosis and treatment led to feelings of altered self-concept, particularly in relation to perceived losses of femininity, sexuality, and gender identity. One participant reflected, *“I got my breast cut off. Yeah…and so it was a sex thing, a sexuality thing for me. It was also a gender thing. I’m a woman and I got my breast cut off”* (FG 4).

Participants also reacted to how their illness was interpreted through socially constructed gender norms and expectations. One participant espoused, *“I’ve stopped using the word mastectomy because that word is genderized. It is a genderized word. … I can see that being an issue with ovarian or needing to get a hysterectomy or those types of things because again it’s… it’s so genderized”* (FG 4).

**Lack of peer support.** Many participants emphasized limited access to peer support following their cancer diagnosis. This gap was particularly pronounced among participants residing in rural, remote, or small/medium population centres. As one participant explained, *“I live … on the west coast of the island, so the amount of group supports for people with cancer are virtually nonexistent”* (FG 3). Lack of access to peer support highlights that geographic location may shape access to supportive care and influence the overall cancer experience.

**Financial toxicity.** Many participants described the substantial financial burden associated with cancer, including difficulty managing the costs of medications, medical expenses, and ongoing care needs. One participant reflected, *“The financial side was probably the most shocking component to me of all of it, of how expensive it is to have cancer”* (FG 4).

Participants also reported that financial assistance programs, such as Employment Insurance (EI), were inadequate to offset the financial burden associated with cancer and reduced work capacity. Many described burdensome application processes, long waiting periods, and strict eligibility criteria that delayed or limited access to timely financial support.

Financial burden was also evident among participants with private insurance, as coverage frequently fell short of meeting treatment-related costs. One participant described, *“The injection that I took after each chemotherapy was $2000. I took one of those every three weeks… I have a private medical plan which covers 80%, which is great, but 20% of $2000 is still a [..] ton of money when you’re doing it every three weeks”* (FG 4). These accounts underscore the persistent financial challenges experienced by individuals undergoing cancer treatment, even within the context of publicly and privately funded healthcare supports.

#### 3.1.3. Social Support Needs and Recommendations

Beyond describing supportive and unsupportive social experiences associated with cancer diagnosis, participants identified several recommendations to strengthen social support systems (Table 2).

**Improved access to cancer peer support programs.** Participants emphasized the importance of peer connection and support during the cancer journey. One participant shared, “*It’s important too for people to speak with other people who have been through the cancer process because you’re getting the real emotions and the real information”* (FG 1).

**Enhanced financial assistance through government-funded initiatives.** Participants emphasized the need for greater and timely financial support to offset the substantial travel-related costs associated with cancer treatment. While partial reimbursement was available through provincial programs, many participants described delays in accessing funding and noted that existing financial support was insufficient to fully mitigate the financial burden of essential cancer care.

**Public education about impact of cancer.** Participants recommended greater public education to improve understanding of the long-term impact of cancer. One participant reflected, *“People didn’t realize… that the side effects go on forever… I think education, public education [is needed]”* (FG 2).

### 3.2. Workplace Experiences

**Figure 2:**
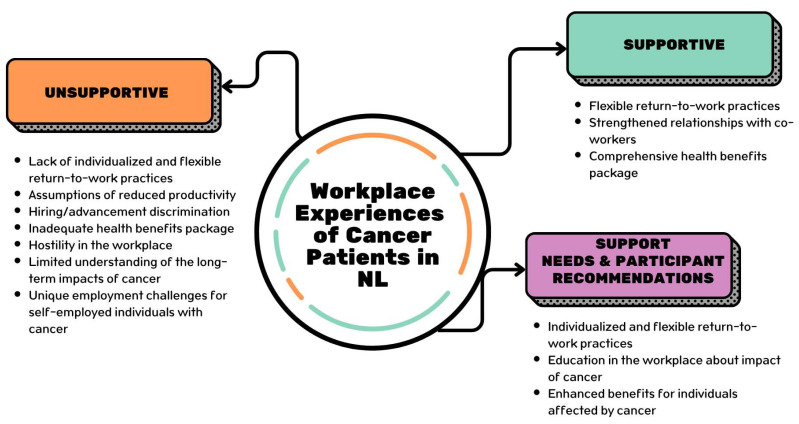
Workplace experiences.

Supportive workplace experiences played an important role in helping individuals navigate the cancer journey. However, inadequate workplace support created considerable challenges and negatively impacted some participants’ ability to manage their health and return to the workplace (Figure 2).

#### 3.2.1. Supportive Workplace Experiences

**Flexible return-to-work practices.** Participants emphasized the significance of individualized and flexible return-to-work practices during and after cancer treatment. For those participants undergoing treatments, the physical toll of these treatments made it challenging to maintain regular work hours, but the ability to work remotely reduced the burden. Individualized and flexible return-to-work practices fostered a sense of control over work–life balance, which was often felt to be lost during the cancer journey. One participant shared, “*Support from the employer…we do care for you… that took a lot of worry away from things and help[ed] me to focus on getting better”* (FG 1).”

**Strengthened relationships with co-workers.** Some participants described strengthened and supportive relationships with their co-workers and employers during their cancer journey. Thoughtful gestures such as cards, messages of encouragement, fundraising efforts were viewed as acts of solidarity and helped participants feel valued and connected to their professional environments. One participant reflected, *“The small group I work with on the [workplace] were fantastic. Two of the girls showed up the day I was getting my head shaved in support”* (WR 2).

**Comprehensive health benefits package.** Participants emphasized that access to a comprehensive health benefits package helped alleviate the substantial burden associated with cancer. Some individuals were able to access mental health services and rehabilitative programs, supporting a holistic recovery. A robust benefits package enabled timely access to follow-up care, supportive programs, and interventions that helped individuals navigate the ongoing challenges of living with or beyond cancer. One participant noted, *“I found the EFAP (which the authors estimated to mean Employee and Family Assistance Program) program really good. I did speak with a psychologist. We get six sessions that were covered through work. I found she talked me off the ledge a few days”* (FG 1).

#### 3.2.2. Unsupportive Workplace Experiences

**Lack of individualized and flexible return-to-work practices**. Some participants experienced a lack of individualized and flexible return-to-work practices. One participant shared, *“They are very by the book and won’t give an inch to their employees*” (FG 1).

Some workplace policies failed to accommodate individualized needs, such as gradual reintegration into work or adjusted work hours to accommodate ongoing cancer treatment. This lack of flexibility often created additional stress, forcing some individuals to either return to work prematurely or take extended leaves of absence, which affected their income and job security. One participant shared, *“I was not even done treatment, probably two or three days, and staffing and the manager calling me to come back into work and I was supposed to be off work for three months post-treatment”* (FG 2).”

**Assumptions of reduced productivity**. Several participants reported experiencing stigmatizing attitudes in the workplace following their cancer diagnosis. They described perceptions by colleagues or employers that they were less capable or less productive. These experiences undermined participants’ sense of belonging and support within the workplace. One participant recalled overhearing co-workers describe them as “*lazy*,” (II-2), an experience that was distressing, despite previously feeling supported and valued within their workplace community.

**Hiring/advancement discrimination.** Some participants reported experiencing negative employment consequences following their cancer diagnosis. One participant shared that their hours of employment were reduced after returning to the workplace. Another participant perceived to lose employment opportunities due to sharing their cancer diagnosis with a potential employer. For example, they shared, *“I got laid off from that [job] and tried to get another job after a couple months after that…. I had applied for jobs, went through interviews…. That probably turned some employers off”* (FG 1). An additional participant shared that when they returned to the workplace, they were encouraged to retire from the company. This participant stated, *“I was called in, right? And the team came up to discuss the retirement, right? Which is not allowed to be discussed to with an employee that’s up to the employee when he wants to go retire, right? But I let…let it go when I shouldn’t of”* (FG 4).

**Inadequate health benefits package**. Limited access to adequate health benefits emerged as a significant concern for many participants. Several participants described insufficient coverage to manage ongoing health needs. One participant shared, *“… physiotherapy, massage therapy, and chiropractic care have a limit of like $500 or something a year or whatever it is…the physiotherapy that comes with your surgery that ends. At a certain time, they can’t keep seeing you forever. And so… I was paying out of pocket then while the, you know, the, the 20% after they covered the 80%, but then when that capped out, then I was paying full for all of those different types of care”* (FG 4). Lack of coverage was particularly challenging for precariously employed individuals. One participant shared, “… *I don’t have insurance because I’m casual”* (FG 1).

**Hostility in the workplace.** Some participants perceived experiences of workplace hostility upon their return to work. One participant reflected, *“…because I have been so unwell, I have had to call in sick quite a bit, …. and has created what I feel to be a hostile work environment”* (WR 3). A hostile work environment exacerbated emotional distress and hindered reintegration into the workforce. A participant shared, *“It has created a work environment in which I feel, anxious, unsupported, and left out”* (WR 3).

**Limited understanding of the long-term impacts of cancer**. Several participants reported that colleagues and supervisors lacked awareness of the persistent physical and psychological consequences of cancer. One participant shared, *“I had both lasting physical and mental impacts, but they didn’t believe it…I really think they thought that I was trying to work the system”* (FG 4). These perceptions reflected a broader lack of understanding regarding the long-term effects of cancer and its treatment and contributed to insufficient workplace support for individuals managing ongoing health challenges.

**Unique employment challenges for self-employed individuals with cancer.** Self-employed participants described unique challenges related to cancer treatment and recovery. One participant explained, *“I’m self employed and, uh, I was off work for 8 months, which was terrifying. My daughter took over a lot of the responsibilities. Now that’s… completely different, I guess from most people who are working for other employers”* (FG 3). In addition to prolonged work absences, self-employed individuals reported a lack of access to employer-sponsored health benefits and formal support systems. A participant noted, *“In private industry, you don’t have that, those benefits or supports”* (FG 3). Many relied heavily on family members to assume essential professional responsibilities, or were compelled to suspend operations temporarily during treatment and recovery. One participant shared, *“I’m self-employed, I lost my vision with my chemo, so I couldn’t work”* (FG 2).

#### 3.2.3. Workplace Support Needs and Recommendations

Beyond describing supportive and unsupportive workplace experiences following a cancer diagnosis, participants identified several recommendations to facilitate survivors’ reintegration into the workplace (Table 2).

**Individualized and flexible return-to-work practices**. Participants emphasized the need for more individualized and flexible return-to-work practices following a cancer diagnosis. As one participant stated, *“I absolutely think more support needs to be present for cancer patients in the workplace. As it’s a debilitating and life altering diagnosis, I think more consideration should be made and accommodation easier accessed… it truly is an experience in which supportive environments are necessary”* (WR 3). This perspective underscores the importance of accessible accommodations and supportive work environments to support employees as they navigate the lasting effects of cancer and reintegrate into the workplace.

**Education in the workplace about the impact of cancer.** Participants recommended the development of educational resources to support workplace reintegration following a cancer diagnosis. One participant suggested that *“…a booklet would benefit the…employee to be able to bring and give it to their manager…just to help educate them as these are the sort of things that this person may need… to re-cover, to be able to go back to their work”* (FG 3). This recommendation highlights the value of structured educational tools to promote employer understanding of the persistent effects of cancer and the need for flexible and individualized workplace accommodations.

**Enhanced benefits for cancer survivors.** Participants emphasized the need for enhanced benefits and more realistic recovery timelines following a cancer diagnosis. As one participant stated, *“The reality is like the whole thought pattern of society needs to change…People need time to recuperate and that means time, not six weeks because the government said six weeks…it’s mentally, physically, financially exhausting”* (II-1). This perspective highlights the inadequacy of fixed leave periods and underscores the importance of comprehensive benefits that acknowledge the prolonged impact of cancer on employee health and wellbeing.

### 3.3. Experiences of Young Adults

**Figure 3:**
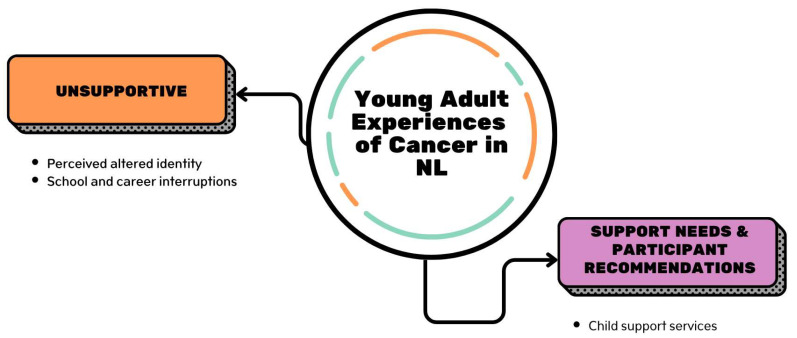
Young adult experiences.

Young adult participants in the study shared distinct experiences during the cancer care journey (Figure 3).

**Perception of altered identity.** One participant described feeling out of place in the cancer care setting due to their young age. They highlighted the challenge of reconciling their identity as a young adult with the realities of living with a chronic illness often perceived to be associated with older age. They explained, “*There’s not many people who are young and diagnosed with cancer, so you can stick out like a sore thumb when you walk through the cancer clinics”* (FG 2).

**School and career interruptions**. Several young adult survivors of cancer reported challenges achieving educational milestones. One participant explained, *“The only thing I had left in my […] degree was to finish my independent practicum… I ended up hospitalized… It was definitely challenging seeing everyone else in my […] class graduate on time”* (FG 2). These experiences highlight the unique challenges young adults face as they strive to balance intensive cancer treatment while pursuing academic and professional aspirations.

#### Young Adult Support Needs and Recommendations

Some young adult participants emphasized the need for greater psychosocial and family-oriented supports during the cancer journey, particularly those with dependent children (Table 2). One participant described the emotional impact of parental illness on children and the lack of support available to families during cancer treatment. One participant shared, *“I’d really like to see supports for people, like my kid. I had young kids at home that were out of school and mamma sick in bed…They could have used phone calls…to be checked up on for sure…so you feel like your kids struggle during your diagnosis”* (FG2).

## 4. Discussion

In this study, we explored the lived social and workplace experiences of cancer survivors. The findings reveal a diverse range of post-diagnosis experiences, spanning both positive and negative impacts, across two key domains of participants’ lives. The participant perspectives contribute to the growing literature on this topic, reflect survivor experiences within the local context, support the generalizability of key literature findings, and generate participant-informed potential solutions for improving cancer survivorship. Within the local context, the findings suggest that there are social, workplace, and financial issues that are experienced by cancer survivors, which are sometimes exacerbated by the geographic dispersion of healthcare- and support-related resources and lack of appropriate information. Findings also have implications for the Canadian policy-making and healthcare/cancer care ecosystem.

### 4.1. Tackling the Financial Toxicity of Cancer Care in Canada

One of the key findings of this study was the substantial financial burden that cancer places on individuals and their families. Financial assistance provided through government programs or workplace benefit packages did not adequately cover daily living expenses, transportation to medical appointments, and loss of income from missed work.

Despite broad cancer care coverage in Canada, many survivors face financial toxicity from both direct costs (such as out-of-pocket expenses for uncovered medications and travel) and indirect costs (including lost income due to time away from work), leading to financial distress [28,29]. Recent estimates suggest that the average lifetime out-of-pocket cost per cancer survivor in Canada is approximately CA$33,000 [36]. The impact of financial toxicity is not distributed equally, and disproportionately affects those with lower socioeconomic status, individuals living in rural, remote, or small-to-medium population centres, and First Nations, Inuit, and Métis peoples [36]. Both self-employed and employed patients/survivors may experience significant income loss [37], contributing to poorer quality of life, compromised cancer care, and worse health outcomes [38].

Almost half of Canadians lack adequate workplace benefits during illness and need to rely on publicly funded programs such as the Employment Insurance Sick Benefits (EI-SB) and Canadian Pension Plan Disability (CPP-D) benefits [28,29]. The EI-SB program is a temporary income replacement program that only provides 26 weeks of coverage and does not adequately address the ongoing financial burden of living with cancer. Consistent with the recommendations of Sayani et al. [28] and Wood and Murphy [29], our results also suggest that the Canadian government may consider extending the duration of EI-SBs to better correspond with the typical duration of cancer treatment.

Eligibility criteria for financial assistance programs in Canada are notably stringent and often leave individuals, including cancer survivors, without access to timely or adequate financial support. For instance, to qualify for EI-SB, applicants must demonstrate that they have accumulated at least 600 h of insurable employment within the preceding 52 weeks [28]. This requirement may be unattainable for precariously employed workers. Similar restrictions exist with the CPP-D benefit, which is designed to provide financial support to individuals with a severe and prolonged disability [28]. As many types of cancer are deemed to be treatable, individuals diagnosed with cancer are often disqualified from receiving CPP-D benefit support. Legislative reform is needed to reduce the financial burden of cancer in Canada. More specifically, as recommended by others [28,29], we also suggest that the Canadian government consider lowering the EI-SB eligibility threshold and recognizing cancer as a “severe and prolonged” disability when it significantly impairs an individual’s ability to work and perform daily activities.

Embedding routine financial toxicity screening into standard oncology care may help mitigate the growing financial strain experienced by cancer survivors. Many oncologists report feeling uneasy and insufficiently prepared to discuss the financial implications of cancer treatment with their patients [39]. Consequently, these essential conversations are often avoided, leaving survivors without the necessary support to manage the significant costs and economic challenges associated with their care [39]. Implementing validated tools like the Comprehensive Score for Financial Toxicity (COST) may enable early detection of survivors at high risk of financial distress and ensure timely intervention and prompt referral to financial navigation services [29,30]. As cancer incidence and treatment-related costs continue to rise, demand for timely and accessible financial support will only intensify. Legislative changes such as expanding coverage of outpatient medications and embedded screening tools in oncology care are powerful strategies that may mitigate the financial toxicity of living with and beyond cancer.

### 4.2. Equitable Insurance Coverage for Cancer Survivors

Insurance companies can play an instrumental role in supporting cancer survivors, as they often cover various out-of-pocket expenditures such as outpatient medications. Unfortunately, many cancer survivors endure financial discrimination when trying to use insurance coverage. These practices significantly limit access to necessary financial protections and exacerbate the economic burden placed on survivors.

The importance, as well as the inadequacy, of insurance coverage during and after cancer was evident in participants’ responses. For example, participants reported that insurance plans did not cover all expenses, that premiums were often unaffordable following a cancer diagnosis, and that some individuals were left without access to any insurance coverage.

In Canada, individuals must disclose pre-existing conditions, including cancer, when applying for individual insurance coverage. Consequently, cancer survivors frequently contend not only with the physical and psychological effects of the disease but also with persistent social and financial challenges that can constrain their opportunities [31]. In contrast, several European countries have implemented *right-to-be-forgotten* (RTBF) legislation [31], allowing individuals to withhold personal health information when applying for insurance after a specified period following treatment [31]. Similar legislative reforms in Canada could help protect cancer survivors from insurance-related financial discrimination.

### 4.3. Individualized Return-to-Work Practices for Cancer Survivors

Participants highlighted the importance of supportive workplace practices when returning to work after a cancer diagnosis. Accommodations, such as flexible schedules, remote work options, and understanding employers, promoted wellbeing, financial stability, and a sense of normalcy. In contrast, inflexible workplace policies and limited accommodations made it difficult to successfully transition back to the workplace. These experiences underscore the need for stronger return-to-work policies and practices for cancer survivors in NL and Canada.

Advances in the early detection and treatment of cancer have made it possible for more cancer survivors to return to work than ever before. However, many continue to experience stigma and discrimination, as well as insufficient accommodations, in the workplace [40,41,42]. Dos Santos et al. [43] identified six key areas of workplace support, including: modifications to organizational structures, environmental adjustments, company and public policies, education for employers and employees, multidisciplinary support, and clear communication. Their findings emphasize the need for individualized approaches to address the unique and often long-term needs of cancer survivors, consistent with our findings.

### 4.4. Addressing the Cancer-Related Information Needs of Cancer Survivors

Cancer survivors prefer ongoing, personalized cancer-related information rather than being overwhelmed with an “information dump” at the time of diagnosis [44]. Timely access to evidence-based information is essential to support informed decision-making, effective self-management, and improved overall quality of life throughout survivorship. Consistent with previous work [25,44,45,46,47], our findings highlight a clear need amongst cancer survivors for timely, accurate, and comprehensive information about their disease, prognosis, treatment options, and other ongoing care needs across the survivorship continuum.

Individuals living outside of urban population areas (rural, remote, and small/medium population centres) often experience unmet cancer-related information needs and are more likely than urban residents to report difficulties understanding cancer-related information [48]. Furthermore, information needs vary by cancer type [48], underscoring the importance of information programs tailored to both specific cancer diagnoses and individual survivor needs.

Given the complexity of cancer survivors’ information needs, innovative solutions are needed. As Richards et al. [32] suggested, mobile apps may help address barriers that limit access to reliable and timely cancer-related health information. Mobile apps may not be accessible or practical for all survivors (because of, for example, the need for internet access and smartphones/computers). Further research is needed to evaluate existing tools and develop interventions that reduce information gaps and improve survivorship outcomes across diverse populations. Public education and awareness campaigns may also help address cancer survivors’ ongoing information needs.

### 4.5. Cancer-Associated Stigma in Social and Workplace Environments

Stigma is a social process where individuals possessing an attribute are deemed undesirable and have a “spoiled identity” [49,50]. Cancer-associated stigma has a profound impact on social interactions and leads to increased exposure to discrimination and job insecurity, stigmatizing healthcare interactions and/or delays in seeking medical care [50,51,52,53].

Participants described experiencing stigmatizing and discriminating interactions with family, peers, partners, colleagues, and employers. Our findings are consistent with a previous study demonstrating the existence of cancer-related stigma and discrimination in NL [25]. Assumptions about post-cancer abilities, misconceptions and fear about cancer, physical changes (e.g., as a result of surgery), social norms associated with affected organs, and other factors such as insurance companies’ business models, lack of understanding about cancer and its impact on survivors, all can contribute to labelling/altered identity, stigma or discrimination in cancer [10,25,50,51,52,53,54]. Several of these stigma-driving factors were detected in the study data (e.g., workplace assumptions, insurance company policies, lack of understanding of cancer’s effects). Gender-based stigma [54,55] was also detected by cancer survivors who identified as female when cancers affected body parts traditionally identified with female identity. These findings highlight the need for context-specific, evidence-based interventions aimed at reducing cancer stigma in public and workplace settings in NL. Addressing stigma however seems to be an ongoing challenge. In this regard, although some promising approaches have been identified, their effectiveness remains inconsistent [56]. Overall, further education, research, and involvement of individuals with lived cancer experience are needed to support stigma reduction efforts [52].

### 4.6. Survivorship Support for Young Adult Survivors of Cancer

Although the sample size of young adults in this study was small, and findings should be deemed exploratory, the study identified important aspects of perceived identity, education and career trajectories in this demographic group. As young adulthood is a critical developmental period, a cancer diagnosis may impede academic and career advancements and contribute to enduring financial challenges [9]. These challenges may be compounded by experiences of stigma and discrimination associated with cancer [25].

Young adults with cancer often balance treatment with parenting responsibilities, placing their children at risk of emotional and practical challenges that may be overlooked in standard oncology care. Findings from this study highlight the need for oncology programs to integrate structured, family-centered support services for children of parents or guardians undergoing cancer treatment. Targeted initiatives, such as regular check-ins and developmentally appropriate supports, may help address emotional and practical challenges experienced by children during periods of disrupted routines. Further research is needed to determine the effectiveness of such interventions in supporting child wellbeing and reducing parental psychosocial burden.

To support recovery and long-term wellbeing, young adults living with or beyond cancer require tailored survivorship programs that extend well beyond active treatment. These programs may be adjusted to evolving needs related to medical care, education, career planning, and life skills development [57]. We note that community-based organizations, such as Candlelighters NL [21] and Young Adult Cancer Canada [20], play critical roles in supporting young adult cancer survivors. In addition, peer support groups specifically for young survivors may be beneficial, as most existing support groups generally consist of older adults, making it difficult for young adults to relate and connect [33].

### 4.7. Strengthening the Peer Support Networks

Cancer survivors may experience feelings of isolation, fear, anxiety, loss of identity, and difficulty reintegrating into everyday life after treatment [58]. Participants in this study identified the need for increased access to peer support services in NL. The absence of these supportive care services left many participants navigating the emotional and social complexities of cancer without adequate support.

Evidence suggests that peer support interventions are linked to perceived benefits such as shared experience, informational support, reduced loneliness and increased coping [34,35]. Although some challenges have been reported [35], online cancer peer support groups facilitated by healthcare professionals appear more effective [34]. With these in mind, formal integration of peer support networks into national and provincial cancer care guidelines may improve access and utility for cancer survivors, including for young adult survivors.

### 4.8. Strengths and Limitations

This study has several notable strengths. It is a patient-oriented study that engaged two patient partners who contributed to all stages of the project. Furthermore, efforts were made to ensure inclusive participation by accommodating various methods of engagement when focus group participation was not feasible. The study generated novel insights into the social and workplace experiences of cancer survivors in NL, offering findings that can inform the public, policymakers, healthcare, and labor systems. Virtual FGs, IIs, and WRs each offer unique strengths and limitations for qualitative data collection. While the themes identified across these methods were largely similar, some themes present in FGs were not captured in IIs or WRs. This discrepancy likely reflects the smaller number of participants whose data were collected using these methods, which limited the diversity of experiences compared to the broader and richer insights obtained from the virtual FGs. Since most participants were highly educated, female, and of Caucasian descent, the lived experiences of cancer survivors from diverse socioeconomic, ethnic, and cultural backgrounds were not fully captured. In addition, most participants (40%) were diagnosed with stage I cancer, whereas only one participant (4%) had stage IV disease, which limited representation across varying cancer stages. Similarly, the largest group of representation was breast cancer survivors (28%) and survivors residing in the Eastern Health Zone (64%); hence, the transferability of findings will need further verification. Furthermore, focus group sizes were smaller than intended sizes due to scheduling conflicts. Although smaller group sizes may have reduced the likelihood of achieving data saturation, typically recommended at 6–8 participants per focus group [24], they also allowed for more in-depth individual contributions and richer discussions. Lastly, as in similar studies, the data and themes identified may have been affected by recall bias, self-selection bias, and convenience sampling.

## 5. Conclusions

This study provides insight into the lived social and workplace experiences of cancer survivors in NL, highlighting major themes of financial toxicity, workplace reintegration challenges, stigma, and gaps in survivorship support and information services. While the themes identified are consistent with those reported in other populations, they also reveal how local context shapes access to cancer care, support, and overall survivorship experiences within NL. High-quality cancer survivorship care requires a coordinated and collaborative approach among multiple stakeholders, each playing a crucial role in addressing the complex challenges that survivors face across social and workplace settings. A comprehensive understanding of the diverse experiences and needs of cancer survivors, as presented here, is essential for informing equitable policies and programs that enhance the quality of life and wellbeing of cancer survivors.

## Figures and Tables

**Table 1:** Sociodemographic characteristics of participants.

Variable	Number (%)
**Type of cancer diagnosed**	
Pancreatic	1 (4%)
Testicular	1 (4%)
Mucinous Carcinoma	1 (4%)
Colorectal	3 (12%)
Breast	7 (28%)
Lung	3 (12%)
Cervical	2 (8%)
Nasopharyngeal	1 (4%)
Endometrial	1 (4%)
Thyroid	1 (4%)
Neuroendocrine	1 (4%)
Vulvar	1 (4%)
Prefer not to answer	2 (8%)
**Disease Stage**	
1	10 (40%)
2	1 (4%)
3	2 (8%)
4	1 (4%)
I do not know	4 (16%)
No Reply	7 (28%)
**Time Since Diagnosis**	
<1 Year	3 (12%)
1 Year	6 (24%)
2 Years	3 (12%)
3 Years	6 (24%)
4 Years	4 (16%)
5 Years	3 (12%)
**Age**	
18–29	3 (12%)
40–49	8 (32%)
50–59	6 (24%)
60–69	6 (24%)
70 or older	2 (8%)
**Marital Status**	
Married	11 (44%)
* Common-law	5 (20%)
Single	3 (12%)
Separated/Divorced	4 (16%)
Widowed	2 (8%)
**Sex**	
Male	7 (28%)
Female	18 (72%)
**Gender Identity**	
Male	7 (28%)
Female	18 (72%)
**Education**	
High School Diploma	3 (12%)
Trade or College Diploma	8 (32%)
University, Undergraduate Degree	7 (28%)
University, Graduate Degree	6 (24%)
Prefer not to answer	1 (4%)
**Residence**	
** Rural Area	8 (32%)
Small Population Centre, with a population between 1000 and 29,999	7 (28%)
Medium Population Centre, with a population between 30,000 and 99,999	2 (8%)
Large Urban Population Centre, with a population of 100,000 or more	8 (32%)
**Health Authority Region**	
Eastern	16 (64%)
Central	4 (16%)
Western	4 (16%)
Labrador/Grenfell	0 (0%)
Prefer not to answer	1 (4%)
**Ethnicity**	
White/European	20 (80%)
Mixed Heritage	1 (4%)
Indigenous (First Nations, Inuit, Metis)	1 (4%)
Black-African	1 (4%)
No Reply	2 (8%)
**Annual Household Income**	
20,000–39,999	1 (4%)
40,000–59,999	1 (4%)
60,000–74,999	3 (12%)
75,000–99,999	3 (12%)
100,000–149,999	6 (24%)
150,000 or more	8 (32%)
Prefer not to answer	3 (12%)
**Length of Employment After Diagnosis**	
Sporadic	1 (4%)
Current	16 (64%)
<1 Year	1 (4%)
1 Year to <2 Years	0 (0%)
2 Years to <3 Years	2 (8%)
3 Years to <4 Years	1 (4%)
4 Years to <5 Years	1 (4%)
Prefer not to answer	3 (12%)
**Self-Employed After Diagnosis**	
Yes	3 (12%)
No	21 (84%)
Sometimes	1 (4%)
**Change in Employers After Diagnosis**	
Yes	1 (4%)
No	24 (96%)

Sociodemographic characteristics of the participants. Only the survey items that were completed by the participants are shown. The full survey can be found in Supplementary Document S2. * Includes long-term relationships. ** According to Statistics Canada [18], rural areas include all geographic regions situated outside population centres.

**Table 2:** Participant support needs and recommendations for improvement.

Subthemes	i. Study Findings and ii. Additional Solutions
	SOCIAL ASPECTS
Improved access to cancer peer support programs	i. Early psychosocial peer support, especially for survivors in rural, remote, or small–medium centres, that can help reduce feelings of loneliness and enhance overall wellbeing throughout the cancer journey. ii. Strengthened peer support networks that consider survivor and demographic group needs (such as young adults). Formally recognized and promoted peer support group concept in cancer care guidelines.
Enhanced financial assistance through government-funded initiatives	i. Enhanced financial assistance through government-funded initiatives to help mitigate the considerable economic burden imposed by cancer as well as reduced administrative barriers that prevent accessing financial resources in a timely and efficient manner. ii. Enhanced Employment Insurance Sick Benefits (EI-SB) and Canadian Pension Plan Disability (CPP-D) Benefits programs. Utilization of tools in the clinic (such as the Comprehensive Score for Financial Toxicity; [COST]) to identify survivors at risk and referral to financial navigation services. Legislative initiatives (such as Right-To-Be-Forgotten [RTBF]) legislation that reduces discrimination while accessing insurance.
Education about impact of cancer	i. Strengthened public education initiatives to raise awareness of the often underrecognized, long-term effects of cancer and its treatment, such as physical, emotional, social, and financial impacts.
	WORKPLACE ASPECTS
Individualized and flexible-return-to-work practices	i. Individualized and flexible return-to-work practices that may be instrumental in supporting the transition back into the workplace.
Education about impact of cancer	i. Workplace education and awareness initiatives that inform employees and employers about the short- and long-term effects of cancer to help reduce stigma and discrimination in the workplace.
Enhanced health benefits for cancer survivors	i. Enhanced health benefit programs to adequately support individuals undergoing cancer treatment and recovery, as the existing benefit structures often reflect the needs of short-term illnesses and do not account for the prolonged, complex, and costly nature of cancer care. Expansion of health benefits to include comprehensive coverage for cancer-related treatments, extended supportive services, and mechanisms that reduce out-of-pocket expenses to improve financial security, promote continuity of care, and better address the long-term health needs of individuals throughout the cancer journey.

Solutions presented are based on perspectives by study participants, authors, and literature [28,29,30,31,32,33,34,35].

## Data Availability

Data that support the findings of this study are available from the researchers. However, restrictions apply to the availability of this data, and so data are not publicly available. The data used in this study cannot be made publicly available as patients were not consented to make their data publicly available or accessible. Transcripts and other data collected from the participants may be available upon reasonable request for researchers who meet the criteria for access to confidential data. Permission to obtain the data can be requested from Krista King (krista.king@mun.ca; Sevtap Savas, savas@mun.ca) and Research, Grant, and Contract Services (ris@mun.ca) at Memorial University of Newfoundland, St. John’s, NL, Canada, and the ethics approval shall be obtained from the Health Research Ethics Board (HREB), Ethics Office, Health Research Ethics Authority, Suite 200, 95 Bonaventure Avenue, St. John’s, NL, A1B 2X5, Canada.

## Supplementary Materials

The following supporting information can be downloaded at: https://www.mdpi.com/article/10.3390/curroncol33060356/s1, Supplementary Document S1. The focus group guide [25,42,59,60,61,62,63]. Supplementary Document S2. Sociodemographic survey.

## Abbreviations

The following abbreviations are used in this manuscript:

COST	Comprehensive Score for Financial Toxicity
CPP	Canadian Pension Plan
CPP-D	Canadian Pension Plan Disability
EI	Employment Insurance
EI-SB	Employment Insurance Sick Benefits
FG	Focus Group
II	Individual Interview
NL	Newfoundland and Labrador
RTBF	Right To Be Forgotten
WR	Written Response
