# Supplementary material for: Social and Workplace Experiences of Individuals with a History of Cancer in Newfoundland and Labrador
Source: Curr Oncol. 2026 Jun 13;33(6):356. doi: 10.3390/curroncol33060356 (PMC13298429; doi:10.3390/curroncol33060356)
Supplement: Supplementary file 1 [file curroncol-33-00356-s001.zip › curroncol-4301187-supplementary.pdf]

SUPPLEMENTARY MATERIALS

Supplementary Document 1. Focus Group Guide

(including extra questions for the young patients)

I. Introduction and Warm-Up: 10 minutes

1. Purpose and agenda
2. Consent and Audiotaping information
3. Introductions and ground rules

II. Social interactions and relations

First, we would like to understand your social relationships following your cancer diagnosis.

1. Do you think that your relationships with family, friends and community members are affected by your cancer diagnosis?

[Possible probes – Have your relationships changed in a better or worse way after your cancer diagnosis? Were there relationships that strengthened and got better after your diagnosis? Were there relationships that weakened, damaged, got awkward, or ended because of your diagnosis?]

2. How do you think you were treated after your diagnosis by your family, friends and community members?

[Probes: Was there support by family members, friends, or community members? Were there positive experiences? Were there negative experiences? Were there distancing, mistreatment, or avoidance by family members, friends, or community members?

3. If your relationships changed after your diagnosis, how did this affect you?

[Probes: If your experiences were positive: Did you experience improvements in health or other aspects of your life as a result? Did it change how you approach social interactions and relationships?

If your experiences were negative: did you distance yourself from these individuals? Did you experience health or other issues as a result? Did it change how you approach to social interactions and relationships? What do you think can help prevent or solve these issues?]

4. How do you think cancer patients should be treated by their family members, friends, and community members? What should their interactions be like?

[Probes: Do you think more support is needed by cancer patients? Do you think that different types of support (for example, emotional, financial, and so on) are needed? If so, by whom and how? What should communications with cancer patients be like? Did you experience insensitive or marginalizing communication due to cancer?]

III. Work place relations and experiences

Next, we would like to understand your workplace relationships following the cancer diagnosis. These relationships include all your work-relations, including your team members, people you supervise, coworkers, employers, supervisors/managers, and the company.

1. Do you think that your workplace relationships are affected by your diagnosis?

[Possible probes – Have your workplace relationships changed for the better or worse after your cancer diagnosis? Were there relationships that strengthened and got better after your diagnosis? Were there relationships that weakened, damaged, became awkward, or ended after your diagnosis?]

2. How do you think you were treated by your workplace colleagues and employers after your diagnosis?

[Probes: Was there support by your employer, coworkers, the company, and others in the workplace? Were there positive experiences? Were there negative experiences? Was there distancing, mistreatment, or avoidance by your employer, coworkers, the company, and others in the workplace? Were your workplace accommodation requests addressed? Did you have the same hiring, promotion and other workplace opportunities as prior to your cancer diagnosis?

3. If your workplace relationships changed after your diagnosis, how did this affect you?

[Probes: If your experiences were positive: Did you experience improvements in health, financial, or other aspects of your life as a result? Did it change how you approach work interactions and relationships?

If your experiences were negative: did you distance yourself from these individuals / the workplace? Did you experience health, financial, or other issues as a result? Did it change how you approach work interactions and relationships? What do you think can help prevent or solve these issues?]

4. How do you think cancer patients should be treated by their work-place colleagues and employers?

[Probes: Do you think more support in the workplace is needed? Do you think that different types of support (for example, emotional, financial, and so on) are needed at the workplace? If so, by whom and how? How do you think communications with cancer patients should be in the workplace? Is there insensitive or marginalizing communications in the workplace due to cancer?]

Additional question for young cancer patient focus group

Can you tell us if there are any additional considerations for young patients with cancer that we haven't talked about?

IV. Closing

Participants will be asked if they have any additional comments or questions and encouraged to raise any other issues that may not have come up in the group discussion. They will then be asked to complete a very short demographic survey.

**This guide is informed by prior work [25, 42, 59-63] and reviewed by the patient partners from target patient groups.*

25. Savas, S.; Winsor, M.; Tenkorang, E.Y.; Simmonds, C.; Stuckless, T. Social stigma associated with cancer in the Newfoundland and Labrador population: An exploratory study. *J. Psychosoc. Onc. Res. Pract.* **2023**, *5*. DOI: 10.1097/OR9.0000000000000100

42. Stergiou-Kita, M.; Pritlove, C.; van Eerd, D.; Holness, L.D.; Kirsh, B.; Duncan, A.; Jones, J. The provision of workplace accommodations following cancer: Survivor, provider, and employer perspectives. *J. Cancer Surviv.* **2016**, *10*, 489–504.

59. Parkinson, M.; Maheu, C. Cancer and work. *Can. Oncol. Nurs. J.* **2019**, *29(4),* 258-266.

60. Nowrouzi, B.; Lightfoot. N.; Cote. K.; Watson. R. Workplace support for employees with cancer. *Curr. Oncol*. **2009**, *16(5),*15-22.

61. Robb, K. A.; Simon, A. E.; Miles, A.; Wardle, J. Public perceptions of cancer: a qualitative study of the balance of positive and negative beliefs. *BMJ Open,* **2014***, 4*, e005434-2014–005434.

62. Gupta, A.; Dhillon, P.K.; Govil, J.; Bumb, D.; Dey, S.; Krishnan, S. Multiple stakeholder perspectives on cancer stigma in North India. *Asian Pac J Cancer Prev*. **2015**, *16(14),* 6141–6147.

63. Shim, H.Y.; Shin, J.Y.; Kim, J.H.; Kim, S.Y.; Yang, H.K.; Park, J.H. Negative public attitudes towards cancer survivors returning to work: A nationwide survey in Korea. *Cancer Res Treat*. **2016**, *48(2),* 815–824.

Supplementary Document 2. Sociodemographic Information Survey

What is your name? -------------------------------------

Please provide us your contact information (email address, phone number): -------------------
----

With what cancer were you diagnosed? Please enter the type of cancer in the space below.

- Prefer not to Answer

What was the stage of your disease at the time of diagnosis?

- Stage I
- Stage II
- Stage III
- Stage IV
- I do not know
- Prefer not to Answer

How many years have passed since your cancer diagnosis? If only months have passed, please tell us how many. Please specify whether months or years in the space below.

Number of Years? -------------------------------------
If applicable, number of months? ------------------------------

- Prefer not to Answer

What is your age?

- 18-29
- 30-39
- 40-49
- 50-59
- 60-69
- 70 or older
- Prefer not to Answer

What is your marital status?

○ Single ○ Legally Married ○ Common-law ○ Divorced/Separated ○ Widowed ○ Prefer not to Answer

What was your sex at birth?

○ Male ○ Female ○ Prefer not to Answer

How would you describe your gender?

○ Male ○ Female ○ Non-binary ○ Other, please describe-------------------------

○ Prefer not to Answer

What is your highest level of education?

○ Less than high school
○ High school Diploma
○ Trade or College Diploma
○ University, undergraduate degree
○ University, graduate degree
○ Prefer not to Answer

Do you live in an urban or rural area? All areas outside population centres are defined as rural. Please choose one of the following that best describes your residence:

○ Small population centre, with a population between 1,000 and 29,999
○ Medium population centre, with a population between 30,000 and 99,999
○ Large urban population centre, with a population of 100,000 or more.
○ Rural area
○ Prefer not to Answer

In what health authority region do you live?

- Eastern
- Central
- Western
- Labrador Grenfell
- Prefer not to Answer

Which of the following best describes your race/ethnicity?

- Indigenous (First Nations, Inuit, Metis)
- Latin-American (e.g. Argentinian, Chilean, Cuban)
- Asian – East (e.g. Chinese, Japanese)
- Asian – South (e.g. Indian, Sri Lankan, Indo-Caribbean)
- Asian – South East (e.g. Vietnamese, Filipino)
- Middle Eastern (e.g. Egyptian, Iranian, Israeli, Palestinian)
- White/European (e.g. English, Greek, Italian, Serbian)
- Black – Africa (e.g. Ghanaian, Somalian)
- Black – North American
- Black – Caribbean Region (e.g. Jamaican, Trinidadian, Barbadian)
- Mixed heritage (Please specify) ________________________
- Other(s), please specify ___________________________
- Prefer not to Answer

What is your annual household income?

- Under $20,000
- $ 20,000 - $ 39,999
- $ 40,000 - $ 59.999

- $ 60,000 - $ 74,999
- $ 75,000 - $ 99,999
- $ 100,000 - $ 149,999
- $150,000 or more
- Prefer not to Answer

For how long were you employed after your cancer diagnosis? Please enter it below:

- Prefer not to Answer

Were you self-employed at anytime after your diagnosis?

- Yes
- No
- Sometimes
- Prefer not to Answer

Did you change employers after your cancer diagnosis?

- Yes
- No (including self-employed)
- Prefer not to Answer
